# Atmospheric temperature, water vapour and liquid water path from two microwave radiometers during MOSAiC

**DOI:** 10.1038/s41597-022-01504-1

**Published:** 2022-09-01

**Authors:** Andreas Walbröl, Susanne Crewell, Ronny Engelmann, Emiliano Orlandi, Hannes Griesche, Martin Radenz, Julian Hofer, Dietrich Althausen, Marion Maturilli, Kerstin Ebell

**Affiliations:** 1 Institute for Geophysics and Meteorology, University of Cologne, Cologne, 50969 Germany; 2 Leibniz Institute of Tropospheric Research (TROPOS), Leipzig, 04318 Germany; 3 RPG Radiometer Physics GmbH, Meckenheim, 53340 Germany; 4 Alfred-Wegener-Institut, Helmholtz-Zentrum für Polar- und Meeresforschung, 14473 Potsdam, Germany

**Keywords:** Atmospheric dynamics, Attribution, Research data

## Abstract

The microwave radiometers HATPRO (Humidity and Temperature Profiler) and MiRAC-P (Microwave Radiometer for Arctic Clouds - Passive) continuously measured radiation emitted from the atmosphere throughout the Multidisciplinary drifting Observatory for the Study of the Arctic Climate (MOSAiC) expedition on board the research vessel Polarstern. From the measured brightness temperatures, we have retrieved atmospheric variables using statistical methods in a temporal resolution of 1 s covering October 2019 to October 2020. The integrated water vapour (IWV) is derived individually from both radiometers. In addition, we present the liquid water path (LWP), temperature and absolute humidity profiles from HATPRO. To prove the quality and to estimate uncertainty, the data sets are compared to radiosonde measurements from Polarstern. The comparison shows an extremely good agreement for IWV, with standard deviations of 0.08–0.19 kg m^−2^ (0.39–1.47 kg m^−2^) in dry (moist) situations. The derived profiles of temperature and humidity denote uncertainties of 0.7–1.8 K and 0.6–0.45 gm^−3^ in 0–2 km altitude.

## Abstract (summary)

**Table:** 

Measurement(s)	radio wave radiation • far-infrared radiation
Technology Type(s)	microwave radiometer
Factor Type(s)	brightness temperature
Sample Characteristic - Environment	planetary atmosphere
Sample Characteristic - Location	Arctic Ocean

## Background & Summary

Observations show that the Arctic is warming at a greater rate than the global average, a feature known as Arctic Amplification^1–3^. Complex mechanisms that are not yet fully understood contribute to the enhanced warming. Water vapour is the strongest greenhouse gas and plays a major role in several processes related to the amplification, but yet to an uncertain degree (i.e., downward longwave radiation flux, clouds, precipitation)^3–5^. The enhanced sea ice loss in the Arctic in summer and autumn causes a greater evaporation, increasing the water vapour load of the warmed atmosphere, which can contain more moisture^2,4^. Moist air frequently intrudes into the Arctic, impeding sea ice formation and driving the retreat of the sea ice edge by increased net radiative warming and mechanical forcing^6–8^. Strong moisture transports with a filamentary geometry are called Atmospheric Rivers^9^, where information with a high temporal resolution is needed to capture the water vapour variability.

Within the past decades, a robust increase of moisture has been detected in the Arctic for certain regions and seasons^10–12^. The increase of moisture content enhances the downward longwave radiation flux and therefore contributes to warming. Especially the autumn and winter months in the Barents Sea and Arctic Ocean are affected by positive moisture trends^12^. However, inconsistencies in the moistening trend among reanalyses call for reliable reference data to evaluate them in the data sparse region of the central Arctic. Radiosonde and satellite data are assimilated in reanalyses and therefore not suitable for independent evaluation. Additionally, water vapour estimations from different satellites disagree among each other, partly due to different measurement principles^13^. Despite the accuracy and high vertical resolution of water vapour and temperature profiles from radiosondes, low sampling rates (one to four sondes per day) and the poor spatial coverage of launch sites in the Arctic impede an adequate representation of the water vapour variability. Remote sensing in the microwave spectrum (satellite- or ground-based) is generally less accurate (lower vertical resolution) and faces several difficulties but has the potential to fill the gaps: Microwave radiometers (MWRs) on board polar orbiting satellites can sample the entire Arctic more than once per day even in cloudy conditions but suffer from uncertainties, for example, due to the lack of knowledge of the highly variably sea ice emissivity^14^ and coarse vertical resolution.

Robust reference water vapour data sets are required for process studies and to evaluate reanalyses and satellite products in the Arctic. The Multidisciplinary drifting Observatory for the Study of the Arctic Climate (MOSAiC) expedition^15,16^ from September 2019 to October 2020 offers a unique set of detailed measurements in the central Arctic. During the expedition the research vessel (RV) Polarstern^17^ from the Alfred Wegener Institute, Helmholtz Centre for Polar and Marine Research (AWI) drifted with the sea ice to investigate coupled atmosphere-ice-ocean processes in the central Arctic to ultimately improve climate models. In this data descriptor, we focus on measurements from the two MWRs MiRAC-P (Microwave Radiometer for Arctic Clouds - Passive), a high frequency MWR especially tailored for low water vapour conditions, and HATPRO (Humidity and Temperature Profiler), a standard MWR commonly used for monitoring of integrated water vapour (IWV). The multi-frequency HATPRO also allows for thermodynamic profiling. MWRs are the the only measurement systems to derive the total cloud liquid (liquid water path (LWP)) in all cloud conditions. From the MiRAC-P observations, we only present the IWV but humidity profiling and LWP derivation will be explored in the future.

The data introduced in this descriptor will be the base of upcoming studies within the Transregional Collaborative Research Centre TR 172 “Arctic Amplification: Climate Relevant Atmospheric and Surface Processes, and Feedback Mechanisms (AC)3”^18^ to study the influence of water vapour and its variability on Arctic Amplification. They can support process studies with high quality IWV and LWP, as well as examinations of boundary layer developments with temperature and humidity profiles with a temporal resolution of one second. Furthermore, the data sets can be used as reference for the evaluation of satellite water vapour products and reanalyses.

## Methods

In this section, we describe the two MWRs HATPRO and MiRAC-P and their measuring principles. Both radiometers were manufactured by RPG-Radiometer Physics GmbH (RPG). In the following, the regression for HATPRO and Neural Network for MiRAC-P to derive meteorological quantities from the raw sensor data are elaborated.

### Microwave radiometers on board Polarstern

The RPG HATPRO G5^19^ from the Leibniz Institute of Tropospheric Research (TROPOS) was mounted on the OCEANET-Atmosphere container, which is routinely operated aboard RV Polarstern since 2009 (e.g.^20–22^). Its two receivers measure radiation emitted from atmospheric gases and liquid water in the microwave spectrum as brightness temperatures (TBs) in 14 channels with an absolute accuracy of 0.5 K. The half-power beam-widths of the receivers are in the range 2–4°. Seven of the channels detect radiation at frequencies between 22.24 and 31.4 GHz (K-band, first receiver) and the remaining ones between 51.26 and 58.0 GHz (V-band, second receiver). The lower frequency band lies along the wing of a weak rotational water vapour absorption line at 22.24 GHz. Channels further away from the absorption line feature lower opacities and are therefore associated with the atmospheric window (e.g., at 31.4 GHz). Henceforth, these channels will be referred to as window channels. Despite the proximity of the 22.24 GHz channel to the water vapour absorption line, the opacity is still quite low so that radiance from all tropospheric layers contribute to the recorded signal (TBs are in the range of 10–40 K). Coarse water vapour profiles can be derived from the shape of the pressure broadened water vapour absorption line. Löhnert *et al*.^23^ found that 1 to 3 independent pieces of information (degrees of freedom) could be resolved in a central European and humid tropical climate. The emission of liquid water is more prominent in window channels and increases with frequency in the microwave spectrum^24,25^. Signals from ice clouds can be neglected because they are transparent in the range of HATPRO frequencies. Apart from humidity profiles, we use the K-band TBs to derive the integrated water vapour (IWV) and liquid water path (LWP). The higher frequency band covers a wing of the oxygen absorption complex at 60 GHz, allowing for temperature profile retrievals because the vertical distribution of the well-mixed oxygen is known^25^. Channels close to the absorption line (58 GHz) feature a high opacity, sensing the radiation emitted from oxygen in the vicinity of the instrument. About 1 to 4 independent pieces of information can be resolved for temperature profiling^23^, depending on the climate and scanning strategy. Most of the time the instrument operated in zenith mode with the elevation angle remaining at 90.0°. The zenith measurements were carried out with a temporal resolution and integration time of 1 s and were interrupted every 30 minutes for 110 s to perform so-called boundary layer (or elevation) scan, sensing the atmosphere at elevation angles of 5.4, 6.6, 8.4, 11.4, 14.4, 19.2, 30.0, and 90.0°. This elongates the instrument’s line of sight through the atmosphere and therefore increases the sensitivity in the atmospheric boundary layer. With this scanning method, we can derive temperature profiles with improved vertical resolution in the lower troposphere (adding about 2 independent pieces of information) resulting in more distinctly resolved height levels when combined with the zenith mode^23,26^. HATPRO can operate in nearly all weather conditions, except during heavy precipitation. A dry blower keeps the radome dry even during slight precipitation, which is recorded by a simple yes/no sensor. Since the measurements are not reliable when the radome is wet, a rain flag has been applied to the data when necessary. Absolute calibrations, where the receivers point at a built-in target at ambient temperature and a target cooled with liquid nitrogen, need to be performed about every 3 months to ensure the TB accuracy. Additionally, a gain calibration is performed automatically to avoid TB drifts (during MOSAiC, the interval of gain calibrations was 315 seconds).

The University of Cologne’s MiRAC-P^27^ (RPG-LHUMPRO-243-340 G5) is a passive MWR that measures atmospheric radiances as TBs at a temporal resolution of 1 s with six channels along the 183.31 GHz (G-band) water vapour absorption line and two window channels centered at 243 and 340 GHz. It was mounted next to HATPRO during the MOSAiC expedition. The six double-sided G-band channels are located at 183.31 ± 0.6, ±1.5, ±2.5, ±3.5, ±5.0, and ±7.5 GHz and, together with the window channels, can be used to derive IWV, LWP, and humidity profiles. The window channels at 243 and 340 GHz feature much higher opacities than the HATPRO window channels because the water vapour continuum absorption strength increases with frequency in the microwave spectrum^24^. At these frequencies, ice particles in clouds scatter atmospheric radiation causing uncertainties in radiative transfer modelling. The G-band water vapour absorption line is significantly stronger than the 22.24 GHz line and can get saturated if the water vapour load is sufficiently high, making the atmosphere opaque. Then, the TBs in the inner G-band (close to the 183.31 GHz line) are in the range of about 240–280 K, depending on the low-tropospheric temperature and moisture distribution. As it will be pointed out later, we can exploit the different absorption line strengths for a complementary usage of HATPRO and MiRAC-P. All MiRAC-P channels use a double side band heterodyne receiver design and have a half-power beam-width ranging from 1 to 1.3°. The off-axis parabolic mirror allows to point the radiometer to 0–180° elevation for sky view or to the internal ambient temperature calibration target (accuracy 0.2 K). During MOSAiC, MiRAC-P operated in zenith mode only. The measurement noise is below 0.5 K for all channels at one second integration time.

### HATPRO: Retrieval via regression

In order to apply the regression with linear or quadratic terms, an example of the latter is given in Eq. (1), coefficients that map TBs to the desired meteorological quantities (IWV, LWP, absolute humidity and temperature profiles) need to be derived by training (*c*_0_, *c*_1_, and *c*_2_). The IWV of the *k*-th sample in the training data set (N samples in total) is computed by

1 $$IW{V}_{k}={c}_{0}+\mathop{\sum }\limits_{i=1}^{m}\left({c}_{1,i}\cdot T{B}_{k,i}+{c}_{2,i}\cdot T{B}_{k,i}^{2}\right)\quad {\rm{with}}\,k=1,\ldots ,{\rm{N}}$$

where *m* is the number of MWR channels considered for this retrieval (7 K-band channels in this case). Here we use coefficients^28^ determined by Nomokonova *et al*.^29^ who applied them to HATPRO data at an Arctic site (Ny-Ålesund, Svalbard). The climatology behind the regression consists of N = 2744 radiosondes launched daily at 12 UTC in Ny-Ålesund covering the period 2006-05-21 to 2017-03-31^30^. The radiosondes have been processed with the GRUAN version 2 algorithm^10,31^. For the regression, simulated TBs from the atmospheric state given by the radiosonde data were obtained with a one-dimensional radiative transfer model that only respects absorption and emission. Since radiosondes cannot measure the liquid water content of clouds, a simple cloud model was applied. Following Karstens *et al*.^32^, a liquid cloud is detected when the relative humidity in a height layer is greater than 95% for temperatures above 253.15 K and the liquid water content is computed with a modified adiabatic approach. Ice clouds are transparent at HATPRO frequencies and therefore not taken into account. The radiative transfer model follows Rosenkranz^33^ for oxygen absorption, Ellison^34^ for liquid cloud absorption, Turner *et al*.^35^ for water vapour continuum absorption, Rüeger^36^ for air mass corrections, and Liljegren *et al*.^37^ for the water vapour line width modelling. Random numbers with a normal distribution multiplied by 0.5 have been added to the TBs to imitate instrument noise with a strength of 0.5 K to match the instrument specifications given by RPG. For the retrievals of IWV, LWP, absolute humidity and temperature profiles from HATPRO’s zenith operation mode, the regression includes both linear and quadratic terms^38^ and only linear terms for the temperature profile based on the boundary layer mode. The evaluation with the test data (identical to training data as in Nomokonova *et al*.^29^) yields overall negligible biases, and standard deviations of 0.37 kg m^−2^ and 14.3 gm^−2^ for IWV and LWP, respectively. The humidity profile standard deviation over the entire test data set is 0.65 gm^−3^ at the surface and decreases to 0.17 gm^−3^ at 5 km altitude. Temperature profiles retrieved from zenith and elevation mode feature the lowest standard deviation at low altitudes, e.g. 250 m (1.5 K) and 150 m (1.0 K), respectively, increasing with altitude.

### MiRAC-P: Retrieval via Neural Network

Given the saturated 183 GHz line, the retrieval problem is strongly non-linear for MiRAC-P. Therefore, we developed a Neural Network (NN), which is described following the published script^39^, based on Python’s tensorflow and keras modules to retrieve IWV. The idea of a NN is to process a given input (e.g., TBs) through one or more hidden layers, connected by so-called activation functions, to generate an output (e.g., IWV). We have refrained from using the Ny-Ålesund radiosonde data for the training of the MiRAC-P IWV retrieval because the dry conditions, where the sensitivity of this instrument is best, were not sufficiently represented. Instead, the training and test data consist of the ERA-Interim (ERA-I) reanalysis from the European Centre for Medium-Range Weather Forecasts (ECMWF)^40^ and simulated TBs. The total number of samples is 24835, distributed over 8 virtual stations (certain grid points) north of 84.5° N and a period from 2001-01-01 to 2017-12-31 with data samples at 00, 06, 12, and 18 UTC^41^. A subset of 12 years from the entire data set, which has been provided by the instrument manufacturer RPG, has been randomly selected as training and the remaining 5 years as test data. Each double side band frequency of the simulated G-band TBs has been averaged to be comparable to the measurements of MiRAC-P.

To obtain a more robust result from the training, we performed the training and evaluation (with the test data) 20 times with different random number seeds. The 20 random seeds were obtained by producing a set of 20 random numbers that lie between 0 and 1000 (boundaries have been chosen arbitrarily). At the beginning of the loop, the seeds of numpy’s and tensorflow’s random number generator were set to the random value. This random value affects the choice of training and test years because a permutation of an index ranging over all years (0–16) defines which ones are selected for training and testing. Of the 17 permuted indices, the first 12 (last 5) mark the training (test) years, respectively. For example, the test data can be 2002, 2006, 2007, 2010, 2016 with the remaining years being used for training. Furthermore, the initialization of the weights in the NN is affected by the seed of tensorflow’s random number generator. As for HATPRO, we also added a random Gaussian noise to the synthetic TBs with a strength of 0.75 K for the G-band channels, 4.2 K for the 243 GHz, and 4.5 K for the 340 GHz channel, as recommended by the manufacturer. The higher noise for the two window channels reduced their weights in the retrieval and therefore diminishes the impact of signals from sources other than water vapour, such as cloud liquid emission or radiation scattered at ice particles. In correspondence with RPG, we chose the input vector of the NN to consist of all MiRAC-P TBs of a time step and the cosine and sine of the day of the year as additional information. The input was scaled to a feature range of −3 to 1 using the MinMaxScaler of the sklearn.preprocessing module. The input layer is connected to the only hidden layer, which has 32 nodes, with an exponential activation function. A linear activation function then links the hidden with the output layer, which only consists of the retrieved IWV. All layers are fully connected. The kernels of the layers are initialized with the default Glorot uniform distribution. Similar to a regression approach, the goal of the training procedure is to adapt the weights of the NN to minimize a loss function that evaluates the predicted with the target IWV. In our case, the mean squared error is used as loss function, minimized with the Adam optimizer^42^. The maximum number of training epochs (number of times the entire training data is cycled through) is 100 with a batch size (number of samples to estimate error gradient before weights are updated) of 64.

During the optimization process, the mean squared error of the test data is monitored to avoid overfitting. Once the test loss did not improve for at least 20 epochs, the training was stopped and the weights that resulted in the lowest test loss were saved. As mentioned before, the training procedure was performed with 20 different randomly chosen seeds to assess the robustness of the NN. Hence, we get the mean and spread of the retrieval performance, quantified by the standard deviation (see Eq. (2)), from the test data (0.55 ± 0.03 kg m^−2^). This value is also noted as a comment in each published retrieval file of the MiRAC-P^43^ and computed as the square root of the bias corrected test loss

2 $$\widetilde{\sigma }=\sqrt{\frac{1}{{\rm{N}}}\mathop{\sum }\limits_{k=1}^{{\rm{N}}}{\left(IW{V}_{{\rm{pred}},k}-Bias-\hat{IW{V}_{k}}\right)}^{2}}$$

with $\hat{IW{V}_{k}}$ being the test data and *IWV*_pred,*k*_ the predicted IWV of the *k*-th sample. The bias is the mean difference between the target (in this case, ERA-I) and predicted IWV. After training, the model is applied to the observed TBs from MiRAC-P with the random seed that produced the lowest overall test loss (seed value: 558).

## Data Records

In this section, the data for the retrieval developments, the measured TBs, and retrieved products are presented for both HATPRO and MiRAC-P. The files for the retrieval development^28,30,41^ have been uploaded to Zenodo, while the remaining files^43–46^ have been published on PANGAEA. All data files are in netCDF format and summarized in Table 1.

**Table 1:** Filenames (prefixes of daily files), purpose (content), publication platform, and DOI of the published retrieval development data sets (measured TB and retrieved product data sets).

Filename	Purpose	Published on	DOI
*_nya_rt00*.nc	Retrieval coefficients for HATPRO	Zenodo^28^	10.5281/zenodo.6673886
MOSAiC_hatpro_retrieval_nya_v00.nc	Retrieval development HATPRO	Zenodo^30^	10.5281/zenodo.5741350
MOSAiC_mirac-p_retrieval_pol_v00.nc	Retrieval development MiRAC-P	Zenodo^41^	10.5281/zenodo.5846394
**Prefix**	**Content**	**Published on**	**DOI**
ioppol_tro_mwr00_l1_tb_v01_	HATPRO TBs (zenith mode)	PANGAEA^44^	10.1594/PANGAEA.941356
ioppol_tro_mwrBL00_l1_tb_v01_	HATPRO TBs (boundary layer mode)	PANGAEA^44^	10.1594/PANGAEA.941356
MOSAiC_uoc_lhumpro-243-340_l1_tb_v01_	MiRAC-P TBs	PANGAEA^46^	10.1594/PANGAEA.941407
ioppol_tro_mwr00_l2_clwvi_v01_	HATPRO LWP	PANGAEA^45^	10.1594/PANGAEA.941389
ioppol_tro_mwr00_l2_hua_v01_	HATPRO absolute humidity profile	PANGAEA^45^	10.1594/PANGAEA.941389
ioppol_tro_mwr00_l2_prw_v01_	HATPRO IWV	PANGAEA^45^	10.1594/PANGAEA.941389
ioppol_tro_mwr00_l2_ta_v01_	HATPRO temperature profiles (zenith mode)	PANGAEA^45^	10.1594/PANGAEA.941389
ioppol_tro_mwrBL00_l2_ta_v01_	HATPRO temperature profiles (boundary layer mode)	PANGAEA^45^	10.1594/PANGAEA.941389
MOSAiC_uoc_lhumpro-243-340_l2_prw_v01_	MiRAC-P IWV	PANGAEA^43^	10.1594/PANGAEA.941470

Behind the prefixes of the daily files the year (yyyy), month (mm), day (dd), hour (HH), minute (MM), and second (SS) are noted as follows: yyyymmddHHMMSS.nc.

The retrieval training data for HATPRO^30^ consists of one file that contains the entire training and test data for the retrieval of temperature (variable name in the file: ta) and humidity (hua) profiles, IWV (prw), and LWP (clwvi) from TBs (tb) measured by HATPRO. The data set is composed of meteorological observations from radiosondes, and simulated TBs. Elevation angles (ele) lower than 90° are only needed for the boundary layer temperature profile. Nomokonova *et al*.^29^ created the regression coefficients^28^ for zenith temperature (tze) and humidity (hze) profiles, boundary layer temperature (tel) profiles, and for IWV (iwv) and LWP (lwp) with this training data set.

The retrieval training data for MiRAC-P^41^ has been provided by the manufacturer RPG and consists of one file that contains the entire training and test data for the retrieval of IWV (prw) from TBs (tb) measured by the MiRAC-P. The sine and cosine of the day of the year, computed from the time variable, are also included. The outline of the data set has been given in the previous section.

The HATPRO TB data set^44^ contains daily files of atmospheric radiance measured as TBs (tb) during zenith (file name contains mwr00) and elevation (file name contains mwrBL00) mode. The retrieved products from HATPRO TBs include daily files of IWV (prw), LWP (clwvi), temperature (ta) and humidity (hua) profiles^45^. Temperature profiles have been retrieved from both zenith (filename contains mwr00) and elevation (filename contains mwrBL00) modes. The uncertainties of the variables are denoted by the expected standard error (prw_err, clwvi_err, hua_err, ta_err). The measured and retrieved data cover the period 2019-10-19 to 2020-10-02. Flag values indicate the quality of the data. The latitude and longitude coordinates of both instruments have been taken from RV Polarstern track data^47–51^.

The MiRAC-P TB data set^46^ is likewise structured as daily files of atmospheric radiation measured as TBs (tb). The TBs of the double side band frequencies (G-band) are averaged and labeled with the upper part of the band (e.g., 190.81 GHz instead of 183.31 ± 7.5 GHz). Similar to the training data^41^, the sine and cosine of the day of the year are included for the NN retrieval. The retrieved IWV (prw)^43^ from MiRAC-P TBs is also compiled into daily files. The IWV uncertainty computed from the retrieval test data is noted as a comment to the retrieved variable and is also given in three categories (dry: [0,5), intermediate: [5,10), moist: [10,100) kg m^−2^).

## Technical Validation

**Fig. 1:**
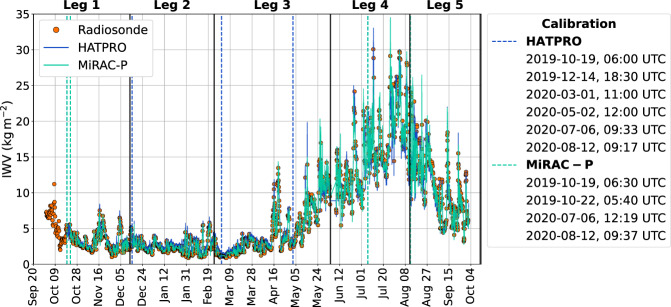
IWV time series from radiosondes (orange circles), HATPRO (blue), and MiRAC-P (cyan) covering the entire MOSAiC expedition (2019-09-20–2020-10-12). A 5-minute running mean has been applied to HATPRO and MiRAC-P data for smoothing. The calibration times of the MWRs are indicated as dashed vertical lines in their respective colours with the exact times noted in the legend. The MOSAiC legs are marked as black vertical lines.

**Fig. 2:**
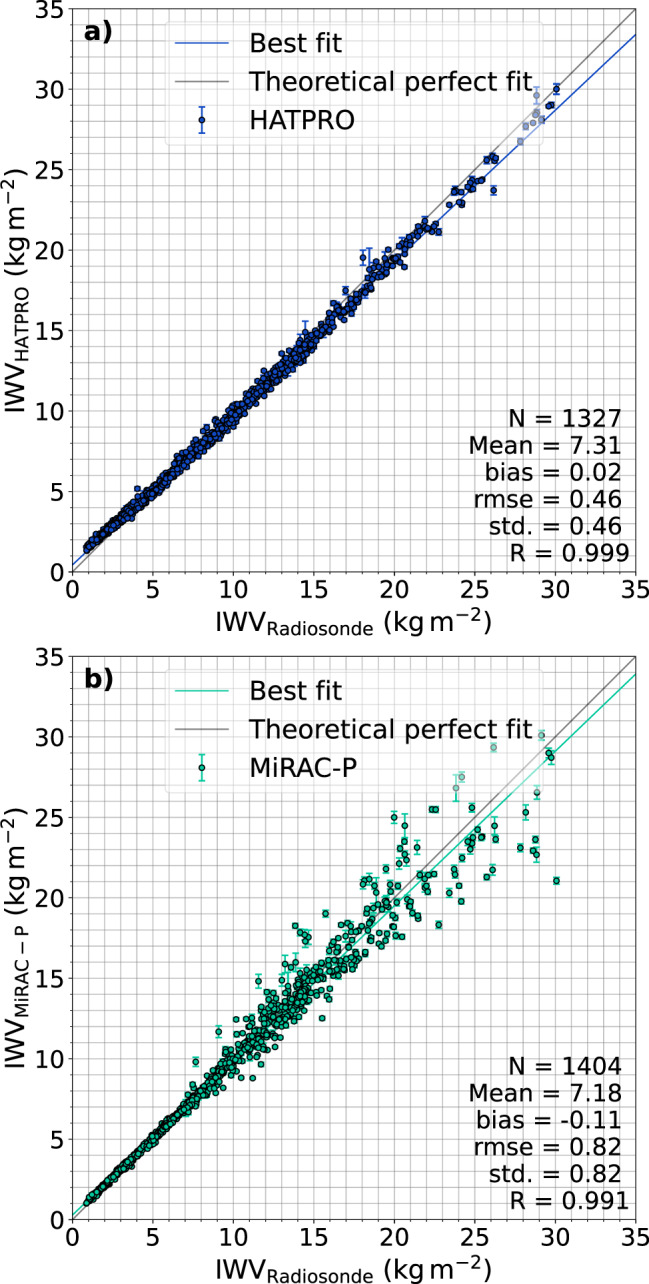
Comparison of the IWV from MOSAiC radiosondes (see text for details) with retrieved IWV from HATPRO (**a**) and MiRAC-P (**b**). The MWR data has been averaged over 15 minutes starting from each radiosonde launch time. The error bars denote the standard deviations of the 15-minute periods. A linear fit has been determined for both radiometers (coloured solid line) and a perfect fit is provided for orientation. Additionally, the number of samples (N), mean, bias, root mean squared error (RMSE), standard deviation (std.), and Pearson correlation coefficient (R) are given.

**Fig. 3:**
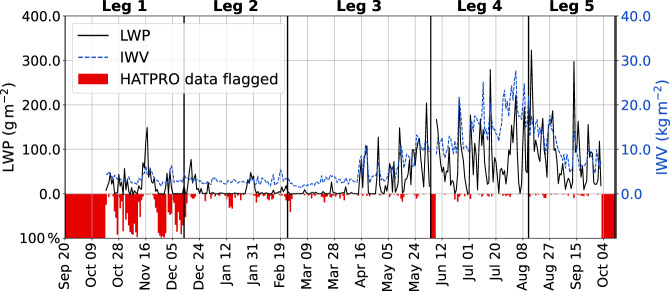
Time series of daily mean LWP (black) and IWV (blue dashed) values retrieved from HATPRO TBs for the entire MOSAiC period. The HATPRO data availability (red bars) shows the fraction of good quality (flag = 0) to the total number of data points on that day. A full red bar reaching from 0 to 100% means that no data without a set flag is available on that day. The absence of red bars implies 100% data availability with high quality.

**Fig. 4:**
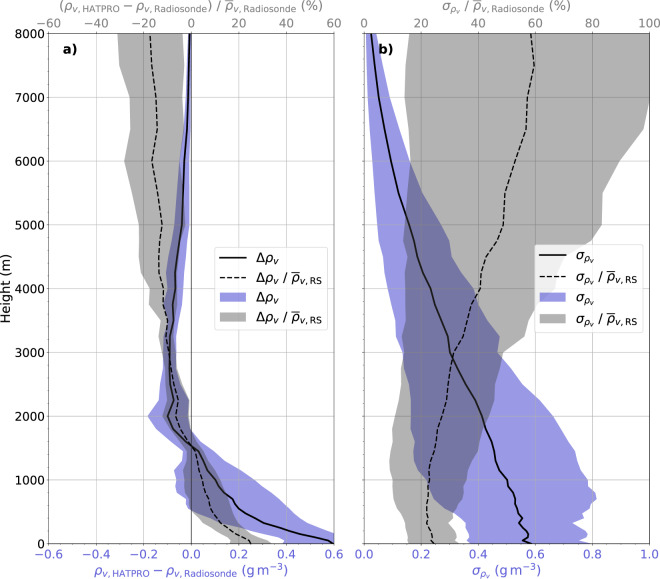
Bias (**a**) and standard deviation (*σ*) (**b**) of absolute humidity (*ρ*_*v*_) profiles between radiosondes (RS) and HATPRO in absolute (solid) and relative (dashed) terms. Shading in blue (grey) indicates the variability over the MOSAiC legs as standard deviation in absolute (relative) terms (see text for details). The relative terms have been normed with the mean radiosonde absolute humidity. HATPRO data has been averaged over 15 minutes starting from radiosonde launch times.

**Fig. 5:**
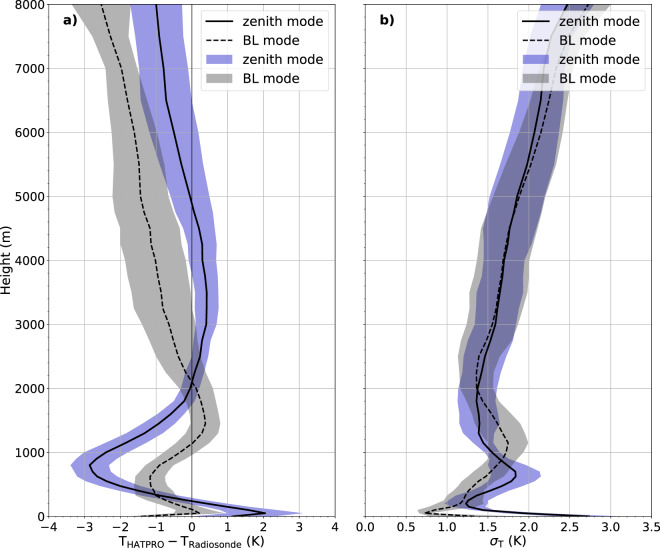
Bias (**a**) and standard deviation (*σ*) (**b**) of temperature (T) profiles between radiosondes (RS) and HATPRO zenith (solid) and boundary layer (BL, dashed) modes. Shading in blue (grey) indicates the variability over the MOSAiC legs as standard deviation for the zenith (boundary layer) mode (as in Fig. 4). HATPRO zenith measurements have been averaged over 15 minutes starting from radiosonde launch times while the boundary layer scans range ±30 minutes around radiosonde launch times due to the lower sampling rate.

In this section, we first discuss the accuracy of TBs and subsequently demonstrate the quality of the derived products — IWV, LWP, absolute humidity and temperature profiles — by comparing them, where possible, to radiosonde observations. Since there is no direct measurement for LWP, we refer to past studies that show the quality of LWP derived from HATPRO^19,52^. Additionally, we compare our LWP with that from the Atmospheric Radiation Measurement (ARM) research facility MWR. The codes to analyze the derived products and generate Figs. 1–5 are openly available^39^ (see also Table 3).

The retrieved temperature and humidity profiles, as well as the IWV, will be compared with radiosonde measurements that have been gathered during the MOSAiC expedition^53^. The radiosondes have been launched from RV Polarstern at least four times per day. We have converted the relative humidity to specific and absolute humidity by using the saturation water vapour pressure method suggested by Hyland and Wexler^54^. Then, we integrated the specific humidity over the pressure levels and divided by the standard gravitational acceleration to obtain IWV. For the comparison of the temperature and humidity profiles with HATPRO, we interpolated each radiosonde onto the height grid of HATPRO profiles. Radiosondes that did not reach at least 10 km altitude and that contained missing values have been rejected in the analysis (23 out of 1522). Drifts of the radiosondes with wind and uncertainties of the temperature, relative humidity, and pressure sensors, which are 0.2–0.4 K, 3–4%, and 0.6–1.0 hPa, respectively^53^, are error sources for the comparison with MWR data.

### Brightness temperatures

Before the retrievals are applied, the quality of the measured TBs was checked following the procedure suggested by Löhnert *et al*.^23^. This involved the flagging of time steps when the rain flag is set, when the sun is within ±7° (elevation and azimuth) of the line of sight of the instrument, when TBs exceed the range 2.7–330.0 K, and when a receiver sanity check fails. The receiver sanity check is based on status flags of an internal procedure implemented by the manufacturer RPG in the housekeeping files of the MWRs, respecting also the receiver stability. Besides automated checks, a manual inspection of the TB data was performed to flag those time steps that show obvious artifacts not related to atmospheric signals (i.e., the crane at the bow of RV Polarstern causing sudden leaps in the TBs). In the following examinations, only time steps with good quality (flag = 0 or *nan*) have been used. The dates when the MWRs were calibrated with liquid nitrogen^19^, to ensure the absolute accuracy of the TBs, are given in Fig. 1. On 2019-10-19, 06:30 UTC, the first calibration of MiRAC-P was carried out but yielded values that differed significantly from previous tests or expectations because the calibration integration time exceeded the maximum value supported by the software. Therefore, the calibration was repeated on 2019-10-22, 05:40 UTC. The MiRAC-P did not require as many calibrations as HATPRO because it showed a negligible drift of TBs over time whereas HATPRO is a standardized instrument recommended to be calibrated every 3 months^19^. Slight jumps in the retrieved data can be found around calibration times. For example, the most noticeable and concurrently the highest absolute jump in IWV is 0.3 kg m^−2^ on 2020-03-01, 11:00 UTC in the HATPRO data.

### Derived products

After applying the retrieval algorithms, the meteorological quantities were inspected whether or not they lie within a reasonable range. LWP must be within [−200,3000] gm^−2^, IWV in [0.0,100.0] kg m^−2^, temperature in [180.0,330.0] K, and absolute humidity in [−0.5,30] gm^−3^, otherwise a flag value was set. The lower end of the thresholds for LWP and absolute humidity are chosen to respect slightly negative values that might result from the regression. For LWP, a further processing step is done. Potential offsets in LWP can be partly corrected using a clear-sky offset correction. Under clear-sky, i.e. here liquid-free, conditions, the LWP should be zero. To determine if a scene is liquid-free, the standard deviation of LWP within a 2-min time interval was analyzed. If this value is below a certain threshold, we assume that no liquid occurs. The threshold depends on the instrument and climate of the location. Based on visual inspection of the derived LWP and also cloud radar reflectivity, the best offset correction was achieved with a LWP standard deviation threshold of 1.5 gm^−2^ for almost the entire MOSAiC period. Only on 2020-07-10, −11, and −12 we used 0.9 gm^−2^ because the other value resulted in highly negative LWP. If all 2-min intervals within a 20-min time window indicate liquid-free conditions, the mean value of the retrieved LWP is calculated and subtracted from the original values. For cloudy periods, the estimated offset values during clear-sky periods are linearly interpolated and subtracted from the retrieved LWP.

#### Integrated water vapour

The MOSAiC expedition gave the opportunity for high quality water vapour measurements in the central Arctic for an entire year. This allows to capture the vast contrasts between winter (polar night) and summer (polar day). The contrast is nicely reflected in the IWV time series over all five MOSAiC expedition legs (measurement periods with a certain scientific crew) from both MWRs and the radiosonde data (see Fig. 1). In winter, the net outgoing longwave radiation and missing energy input from the sun can cause temperatures to drop to values below −35 °C^55^ making the air extremely dry due to the Clausius-Clapeyron relation. IWV is frequently below 4 kg m^−2^ from December 2019 to mid-April 2020 and can even be as low as 0.8 kg m^−2^ (February and March 2020). Only during occasional storms the IWV peaks above 5 kg m^−2^ (i.e., mid February 2020). As soon as the melt season commences in late spring (May 2020), the IWV shows much higher values (up to 30 kg m^−2^) and a greater variability on synoptic scales (few days). In general, when merely considering the time series, all three data sets capture the extreme differences between winter and summer very well, proving the capability of the MWRs to capture the full range of IWV conditions. During synoptic events, such as cold air outbreaks or moist air intrusions, the benefit of the MWRs compared to the radiosondes is obvious. The MWRs capture the temporal evolution of IWV much better with their resolution of 1 s than the radiosondes, which were mainly launched four times a day during the expedition. IWV variabilities, gradients and extreme values, of which the latter might be missed by radiosondes, can be resolved at time scales of minutes or even seconds^56^. The extraordinarily strong moist air intrusion that occurred in mid-April 2020 is shown in greater detail in the Usage Notes as an example of the retrieved products.

To analyze the differences between radiosondes and the MWRs, the data sets are displayed against each other in Fig. 2. For the comparison, the MWR data has been averaged over 15 minutes starting from the radiosonde launch times. The standard deviations of these 15-minute periods are shown as error bars and indicate the noise but also the variability of the retrieved products. When we omit radiosondes that failed the quality check (as noted above) and MWR data where the flag value does not indicate good quality, a total of 1327 (1404) radiosonde launches are left to compare to HATPRO (MiRAC-P) data. From Fig. 2, the complementary nature of HATPRO and MiRAC-P is visible. The MiRAC-P agrees better with radiosondes in dry conditions compared to HATPRO, which indicates the superior sensitivity of the strong G-band water vapour absorption line. To point out the complementary precision of MiRAC-P and HATPRO, Table 2 summarizes the standard deviations (computed as in Eq. (2), but with $\hat{IW{V}_{k}}$ representing the radiosonde and *IWV*_pred,*k*_ the MWR), biases, and root mean squared errors with respect to the radiosonde IWV for three IWV classes (dry: [0,5), intermediate: [5,10), moist: [10,100) kg m^−2^). On average, HATPRO shows a bias of 0.35 kg m^−2^ for IWV smaller than 5 kg m^−2^ (see Table 2). Below 3.5 kg m^−2^, the bias ranges from 0.25 to 0.75 kg m^−2^. Here, higher biases occur in the drier conditions (lower IWV). Due to the superior sensitivity of MiRAC-P in dry conditions, a bias nearly three times lower (0.12 kg m^−2^ instead of 0.35 kg m^−2^) can be seen for IWV smaller than 5 kg m^−2^. In the dry regime, the MiRAC-P features a considerably lower standard deviation (0.08 kg m^−2^) than HATPRO, which shows 0.19 kg m^−2^. Even in the range 5–10 kg m^−2^, the majority of the MiRAC-P data denotes differences to the radiosondes within [−0.25, +0.25) kg m^−2^ resulting in a bias of 0.0 kg m^−2^, while the standard deviations of both MWR retrievals are similar (≈0.3 kg m^−2^) in that IWV range. When the IWV is greater than 10 kg m^−2^, the retrieved IWV from MiRAC-P starts to scatter because the atmosphere becomes opaque to the G-band channels close to the absorption line. In other words, these channels become saturated and an increase in IWV does not change the TB any longer (e.g.^57,58^). The higher the IWV, the more channels further away from the absorption line are affected by this saturation effect. The radiative transfer simulations of the training data have shown that the 183.31 ± 7.5 and 243 GHz channels are the only frequencies that can still detect IWV increases through TB changes for IWV above 15 kg m^−2^. But in these frequencies and moist conditions, many TBs map to the same IWV so that no clear relation between the TBs and IWV can be inferred. This could explain the strong scattering of IWV from MiRAC-P when compared to the radiosonde measurements in moist conditions as seen in Fig. 2, resulting in a standard deviation of 1.47 kg m^−2^ (see Table 2). HATPRO shows the opposite behaviour for high IWV, having an uncertainty of 0.39 kg m^−2^, which is almost a factor of 4 lower than the uncertainty of MiRAC-P.

**Table 2:** Root mean squared error (RMSE), bias, standard deviation (*σ*) of IWV between HATPRO or MiRAC-P and radiosondes, divided into three categories of low, intermediate and high moisture load.

IWV range (kg m^−2^)	Instrument	N	RMSE (kg m^−2^)	bias (kg m^−2^)	*σ* (kg m^−2^)
[0,5)	HATPRO	651	0.40	0.35	0.19
	MiRAC-P	730	0.15	0.12	0.08
[5,10)	HATPRO	279	0.33	−0.14	0.29
	MiRAC-P	276	0.35	0.00	0.35
[10,100)	HATPRO	397	0.61	−0.47	0.39
	MiRAC-P	398	1.49	−0.23	1.47

Additionally, the number of samples (N) of the respective subclass and instrument is given.

When considering the entire IWV range, the bias of the MiRAC-P (HATPRO) product is −0.11 kg m^−2^ (0.02 kg m^−2^), with a standard deviation of 0.82 kg m^−2^ (0.46 kg m^−2^). Compared to the Global Navigation Satellite System (GNSS) IWV retrieval performed by Männel *et al*.^59^, who found a bias of 0.08 ± 0.04 kg m^−2^ and a root mean squared error of 1.47 kg m^−2^, the two MWRs yield more precise estimates of IWV, and HATPRO also a higher accuracy, when considering the entire range. The ARM research facility also derived IWV from their two-channel MWR, which was also located onboard RV Polarstern^60^. Their retrieval (MWRRET) combines a statistical and physical approach (Optimal Estimation), that also takes surface observations and radiosonde IWV into account, to generate a best estimate IWV data set (for a detailed description, please see^61^). ARM’s MWR provides a IWV record with a lower temporal resolution (26 seconds on average) and a roughly 20-day long data gap in August 2020. Both the lower resolution and the gap have to be taken into account when comparing their product with ours from HATPRO and MiRAC-P. Reducing the radiosonde and the three MWR data sets to a common time grid, where all quality flags indicate good quality, leaves us with 813 radiosondes to compare. The MWRRET best estimate yields a bias (standard deviation) of −0.21 kg m^−2^ (0.44 kg m^−2^), while our products show −0.01 kg m^−2^ (0.44 kg m^−2^) and −0.08 kg m^−2^ (0.75 kg m^−2^) for HATPRO and MiRAC-P, respectively. Below 5 kg m^−2^ (10 kg m^−2^), the performance of our products is especially good, having a standard deviation of 0.19 kg m^−2^ (0.29 kg m^−2^) and 0.07 kg m^−2^ (0.28 kg m^−2^) for HATPRO and MiRAC-P, respectively, while it is 0.40 kg m^−2^ (0.48 kg m^−2^) for the MWRRET best estimate.

#### Liquid water path

The LWP is an important quantity for the evaluation of reanalyses and radiation balance. HATPRO, MiRAC-P and the two MWRs from the Atmospheric Radiation Measurement research facility (of which the three-channel MWR did not operate during most of the time^16^) are the only instruments onboard RV Polarstern capable of retrieving LWP in all cloud conditions. Throughout the MOSAiC expedition, the LWP features a distinct seasonal variability (see Fig. 3) with seasonally averaged daily mean LWP of 8, 25, 91, and 40 gm^−2^ for winter (December–February), spring (March–May), summer (June–August), and autumn (September–November). Also the variability of the daily mean within a season, computed as seasonal standard deviations of the daily mean LWP, shows an annual cycle with 15, 38, 67, and 49 gm^−2^ for winter, spring, summer, and autumn, respectively. In summer, daily average LWP can exceed 250 gm^−2^. This seasonality was also seen at Ny-Ålesund by Nomokonova *et al*.^29^. Higher values of LWP frequently occur in conjunction with high IWV because the moister air masses tend to generate more or deeper clouds. Former studies have proven the quality of the retrieved LWP, having an uncertainty of merely 14–23 gm^−2 ^^19,52^. In winter, when LWP is frequently within the uncertainty range (see Fig. 3), the LWP estimates must be considered with care. Although retrieval noise might still result in slightly negative LWP, the clear-sky offset correction improved LWP biases. Comparing LWP derived from HATPRO with the best estimate from ARM’s two-channel MWR (MWRRET)^60^, we find that more than 81% of the data values agree within ±17.5 gm^−2^ and 93% within ±27.5 gm^−2^. For the comparison both data sets have been merged onto the same time grid due to differences in temporal resolution and data availability (as for IWV, see above).

The data availability in Fig. 3 shows the fraction of non-flagged (flag = 0 or *nan*) values to the total number of data points of a day. During MOSAiC leg 1 (2019-09-20–2020-12-13), the internal sanity check of HATPRO frequently indicated a problem with the receiver of the V-band channels. The problem did not persist beyond the calibration on 2019-12-14, 18:30 UTC from where on the fraction of flagged values decreased significantly.

#### Humidity profiles

Humidity profiling from HATPRO data is more challenging than estimating the integrated amount because of the low information content (usually 1 to 3 independent pieces of information^23^). The dry conditions of the Arctic and the frequent occurrence of strong vertical gradients and moisture inversions^62–65^ impede it further. The retrieved absolute humidity profile may still contain slightly negative values in high altitudes because of retrieval noise but flags are set for values below −0.5 gm^−3^.

As for the comparison of IWV from HATPRO and radiosondes, the HATPRO data has been averaged over 15 minutes, starting from each radiosonde launch time, to evaluate the retrieved absolute humidity profiles. Systematic differences (bias) are expressed as the mean difference of absolute humidity over time on each height level (Δ*ρ*_*v*_ = ρ_*v*,HATPRO_ − *ρ*_*v*,radiosonde_) in absolute and relative terms (Fig. 4a). The latter has been normalized by the mean absolute humidity from radiosondes after averaging. The standard deviation of absolute humidity with radiosonde data as reference is also given in relative and absolute terms. As above, the relative term of absolute humidity standard deviation shown in Fig. 4b has been computed by normalization with the mean radiosonde absolute humidity after determining the absolute term. While this procedure (normalizing after averaging) may not capture the individual relative differences for each radiosonde (normalizing before averaging), it is sufficient to give an idea of the relative uncertainty of the retrieved humidity profiles. We computed the bias and standard deviation for each MOSAiC leg so that Fig. 4 displays the mean (standard deviation) of these quantities over the legs as black lines (shading).

In the lowest 1.5 km, HATPRO overestimates the absolute humidity with the highest bias (0.6 gm^−3^ or 25%) at the surface. Further above, the bias becomes negative, up to about −0.1 gm^−3^ at 2–3 km height, and approaches zero in the remaining atmospheric column (up to 10 km). This is a typical behaviour when humidity inversions or strong moisture gradients are smoothed out in the retrieved profile. The integrated humidity content (IWV) stays free of bias when a positive bias at the surface is balanced by a negative one in greater heights. In winter, when the humidity is low, the relative bias is usually higher than in summer.

At the surface, the standard deviation is 0.59 gm^−3^ or 25% in absolute or relative terms, respectively (see Fig. 4b). Because of the general decrease of absolute humidity with height, the standard deviation in absolute values also approaches zero with values of 0.41 gm^−3^ at 2 km height and 0.02 gm^−3^ at 8 km height. However, the relative standard deviation increases to 27% and 58% at those heights, respectively. Therefore, above 5 km altitude, when the standard deviation is near 50%, the retrieved profile from HATPRO must be considered with care.

Past studies have found standard deviations of HATPRO-retrieved to radiosonde profiles of 0.9–0.6 gm^−3^ in the lowest 2 km^66,67^, which are slightly higher than those found here (0.59–0.41 gm^−3^). However, their studies were carried out with data in the mid-latitudes, where the water vapour load is much higher. Ebell *et al*.^66^ found a relative uncertainty of 12% in the lowest 2 km, while our analysis shows 22–27%. To reduce humidity profile uncertainties and improve the information content, we are thus working on a synergetic retrieval of IWV and humidity profiles combining the measurements of both HATPRO and MiRAC-P.

#### Temperature profiles

HATPRO temperature profiles from the zenith mode have been averaged the same way as absolute humidity profiles for the comparison with radiosonde data. The measurements in boundary layer mode, performed only once every 30 minutes, were averaged over ±30 minutes around radiosonde launch times due to the lower sampling rate. In Fig. 5, the bias and standard deviation profiles can be seen for both measurement modes. Shading indicates the variability over the MOSAiC legs as described in the previous section and also shown in Fig. 4. In the lowest 800 m, both modes show biases that quickly change with height (zenith: 1.2 to 2.0 to −2.8 K, elevation: −1.4 to 0.2 to −1.2 K). Radiative cooling over sea ice causes strong surface temperature inversions persisting almost throughout the entire winter^10,68,69^. The presence of low clouds may also generate inversions due to cloud top cooling^64,70^. In summer, when the solar energy is used to melt the snow and sea ice^71^, temperatures remain close to the freezing point at the surface despite the possible presence of warmer air masses aloft. Therefore temperature inversions are weaker and located at greater heights in summer^68^. Although the rapid changes of temperature over a few hundred metres below 1 km altitude cannot be resolved by HATPRO, the boundary layer mode denotes lower biases (standard deviations) than the zenith mode by up to 2 K (0.5 K). The standard deviation in 0–2 km height is 0.7–1.7 K (1.2–2.7 K) for the boundary layer (zenith) mode and therefore higher than the values found by Löhnert and Maier^26^ (0.5–1.4 K). This is likely due to the nearly permanent presence of inversions in the low Arctic troposphere. The switching signs of the biases up to 2 km height suggest that the inversions are smoothed out with a warm bias at their lower and a cold bias at their upper end for the zenith mode. Averaged over height and time, the boundary layer mode features a consistent cold bias.

Above 2 km altitude, the bias of the zenith mode is smaller compared to the boundary layer scan (−1.0 to 0.4 K vs. −2.6 to 0.0 K). Up to about 4.5 km altitude, the standard deviations are similar, increasing from 1.4 to 1.8 K (see Fig. 5). In greater heights, the standard deviation of the zenith temperature profile increases (up to 2.5 K in 8 km) because information comes from more height levels at once (broader weighting functions) providing less distinctly resolved levels in these altitudes. At heights between 2 and 4 km, we found similar uncertainties as in Löhnert and Maier^26^, who identified standard deviations of 1.4–1.7 K.

## Usage Notes

In this section, we give some recommendations on handling the MWR data and provide an example of their capabilities for using them in a case study. When importing either the TB data or the retrieved products, data where the flag is not 0 or *nan* must be considered with care (remark: Python library xarray converts the good quality indicator to *nan* while netCDF4 leaves it at the fill value 0). Importing the zenith temperature and humidity profiles from HATPRO over a long time period might result in large memory usage when using a library like numpy (one month of zenith temperature profiles results in roughly 100 million data points of type float32). Therefore, downsampling or the usage of a library like xarray, which compresses the data, is highly recommended. The comparison of the HATPRO temperature and humidity profiles with radiosonde measurements is just one example of downsampling. Regarding the IWV, MiRAC-P should be used for values lower than 5 kg m^−2^ and HATPRO for values greater than 10 kg m^−2^ to optimally exploit the data sets. A transition zone from MiRAC-P to HATPRO IWV could be established in the range 5–10 kg m^−2^ where both instruments work similarly well. For temperature profiles, a combination of zenith and boundary layer modes (0–2 km: boundary layer mode, 2–10 km: zenith mode) yields the best estimate.

The MOSAiC data policy requires a moratorium for the TB and retrieved data products until 2023-01-01. Only researchers that are a part of the MOSAiC community will have access before that date.

### Moist air intrusion case

**Fig. 6:**
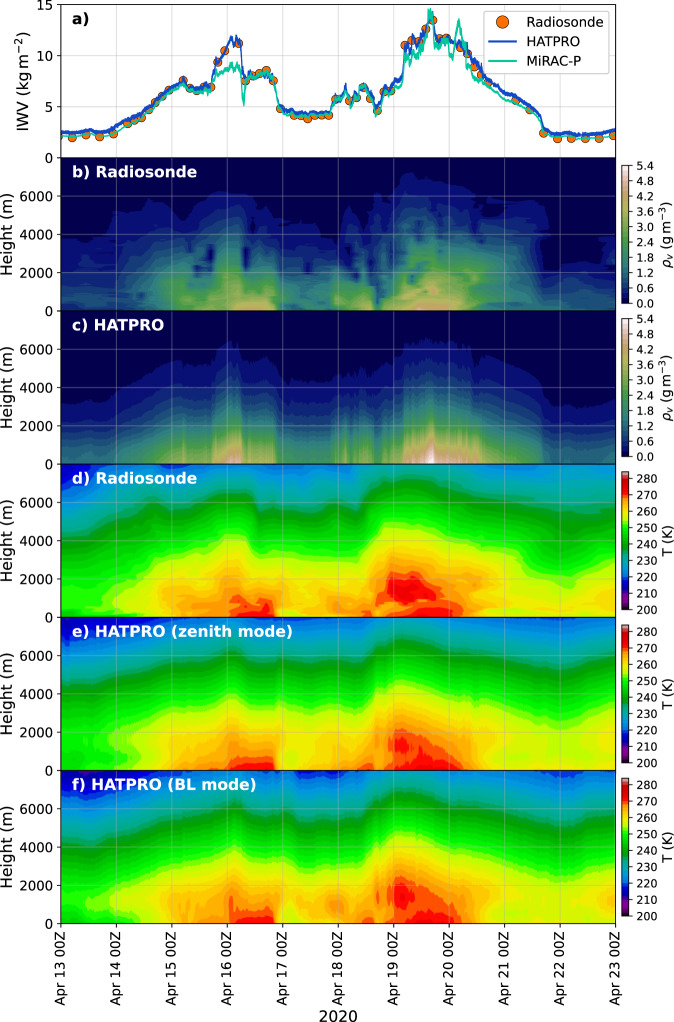
Overview of a moist air intrusion case during the MOSAiC expedition from 13^th^ to 23^rd^ April 2020 showing the IWV as in Fig. 1 (**a**), absolute humidity (*ρ*_*v*_) profiles from radiosondes (**b**) and HATPRO (**c**), as well as temperature (T) profiles from radiosondes (**d**), HATPRO zenith (**e**) and boundary layer (BL) (**f**) modes. HATPRO humidity and zenith temperature profiles have been resampled to one-minute averages.

During the MOSAiC expedition a record breaking moist air intrusion was captured in April 2020^55^. The codes used for importing, processing, and visualizing the data as seen in Fig. 6 are published on Zenodo^39^. Time stamps where the flag indicates bad quality have been filtered out and the radiosonde data was interpolated to a height grid with 5 m resolution, ranging from 0 to 15 km altitude. We have resampled HATPRO humidity and zenith temperature profiles to one-minute averages to reduce the number of data points. In Fig. 6, the time series of IWV, temperature and humidity profiles of the moist air intrusion case are shown. Since this was considered an intensive observation period due to the anomalous conditions, radiosondes were launched up to 7 times per day, which translates to a temporal resolution of 3 to 4 hours. However, despite the unusually high number of radiosonde launches, the full variability of IWV cannot be caught as well as with HATPRO and MiRAC-P. It is likely that the radiosonde data missed the maximum of 14.3 kg m^−2^ (HATPRO) IWV on 2020-04-19 by 0.8 kgm^−2^. On that day, the radiosondes also experienced a strong horizontal drift due to high wind speeds, which could explain the discrepancy between HATPRO and radiosondes as well. Additionally, the MWRs detect the steep temporal gradient of the IWV much clearer than the radiosondes (i.e., between 2020-04-19 00 and 06 UTC). In the humidity profiles, the limited vertical resolution and biases at the surface of the HATPRO product are obvious. Even the strong humidity inversions that were detected by the radiosondes from 2020-04-19 00 UTC until 2020-04-21 12 UTC are not resolved. Also lifted layers of dry air with strong humidity inversions at their upper end (e.g., 2020-04-16 08 UTC) cannot be identified in the HATPRO humidity profiles. Below 2 km, small temperature inversions like the one seen in the radiosonde profile on 2020-04-16 are, at least, seen as isothermal layers in the HATPRO temperature profiles. The benefit of the boundary layer over the zenith mode is more distinct on 2020-04-18 00 UTC and 2020-04-19 00 – 12 UTC when the strong temperature inversion in 0–2 km height is clearly resolved .

**Table 3:** First block: Neural network retrieval development and application on MiRAC-P TB data^46^, generating the derived product^43^.

Filename	Purpose
NN_retrieval_miracp.py	Neural Network retrieval training and application
data_tools.py	Module containing data analysis routines called by other scripts
import_data.py	Module containing various importer routines called by other scripts
met_tools.py	Module containing meteorological computations (humidity conversion, ...) called by other scripts
my_classes.py	Classes called by other scripts
case_study_overview_mwr_radiosonde.py	Script to generate Fig. 6
mwr_pro_output_add_geoinfo.py	Adding Polarstern track data^47–51^ to HATPRO files
PANGAEA_tab_to_nc.py	Script to convert PANGAEA radiosonde^53^ and Polarstern track data^47–51^ to netCDF format
plot_mwr_level_2a_radiosonde.py	Script to generate Figs. 1–3
plot_mwr_level_2bc_radiosonde.py	Script to generate Figs. 4 and 5

Second block: Auxiliary modules called by other scripts. Third block: Visualization and data processing scripts. The codes are freely available^39^.

## Data Availability

Almost all parts of this study have been coded with Python (version 3.8.10) using the following libraries: tensorflow (2.5.0), keras (2.5.0), numpy (1.17.4 and 1.19.5 (latter for NN retrieval)), sklearn (0.24.2), netCDF4 (1.5.3 and 1.5.7 (latter for NN retrieval)), matplotlib (3.4.3), and xarray (0.18.2). The codes of the NN retrieval, the visualization scripts for the Technical Validation and Usage Notes are openly accessible^39^ and listed in Table 3. The scripts for HATPRO retrievals and processing of TB data of both instruments, written in the programming language IDL, are also available on Github and Zenodo^39^.
